# Effective fetch and relative exposure index maps for the Laurentian Great Lakes

**DOI:** 10.1038/sdata.2018.295

**Published:** 2018-12-18

**Authors:** Lacey A. Mason, Catherine M. Riseng, Andrew J. Layman, Robert Jensen

**Affiliations:** 1 School for Environment and Sustainability, University of Michigan, Ann Arbor, Michigan, USA; 2 Great Lakes Environmental Research Laboratory, National Oceanic and Atmospheric Administration, Ann Arbor, Michigan, USA; 3 Baltimore District, U.S. Army Corps of Engineers, Baltimore, Maryland, USA; 4 Engineer Research and Development Center, U.S. Army Corps of Engineers, Galveston District, Vicksburg, Mississippi, USA

**Keywords:** Physical oceanography, Ecological modelling, Limnology

## Abstract

Wind exposure is a key physical driver of coastal systems in aquatic environments influencing circulation and wave dynamics. A measure of wind exposure is fetch, the distance over which wind can travel across open water. In large lake systems, such as the Laurentian Great Lakes, estimating fetch has proven to be difficult due to their vast size and complex topobathymetry. Here we describe the development of two spatially discrete indicators of exposure to provide a more accurate indicator of the influence of wind exposure in the nearshore of the Laurentian Great Lakes. We summarized wind data from offshore buoys and used existing tools to calculate effective fetch and a relative exposure index (effective fetch scaled by mean wind speed) at a 30-m grid cell resolution. We validated these models by comparing our exposure maps to the U.S. Army Corps of Engineers Wave Information Studies models and found general agreement. These exposure maps are available for public download for the years 2004–2014.

## Background & Summary

Wind exposure is a key physical driver of coastal systems in marine^1^, large lake^2^, and inland lake^3^ environments and is an important indicator of the availability and type of coastal habitat^4^. In nearshore areas, wind exposure affects shoreline erosion rates^5^, sediment sorting and resuspension^6^, aquatic organism distributions^7,8^, and thus the suitability of aquatic habitat^9,10^ to support different communities. In the offshore, wind exposure influences currents^11^, thermal stratification^10,12^, upwelling^13^, and lake turnover events^14^. Wind exposure is commonly measured as fetch, the distance over which wind can travel across open water. In large lake systems with variable wind patterns and complex topobathymetry, the ability to incorporate the variability of wind direction and velocity into one index would provide for a more specific approach to quantifying relative wind exposure of a site.

Fetch and exposure of the nearshore to wind and waves has been found to be an important factor in structuring ecosystems and their biological communities from plants^15^ to fish^7^. For example, exposure of the coast influences the types of coastal wetlands that may form and the vegetation community structure within those wetlands^15^. The vegetation communities, in turn, influence the diversity, structure, and productivity of invertebrate^8^, bird, reptile and fish communities that represent the food webs that use those resources^16^. Great Lakes coastal wetlands provide important habitats essential for multiple organisms and life stages. Jude and Pappas^17^ found 113 species of Great Lakes fish use coastal wetlands for spawning, nursery, shelter, or permanent habitat. Realistic calculation of nearshore and coastal exposure will aid in identifying specific locations for restoring and managing coastal wetlands and provide input to guide sampling, monitoring and protection plans for species that use specific coastal wetland types or more exposed habitats.

In large lake systems, such as the Laurentian Great Lakes, estimating an accurate measure of fetch has proved to be difficult due to their vast size^18^ (Great Lakes total surface area = 246,028 km^2^) and complex topobathymetry and wind patterns. The Great Lakes contain over 29,000 islands^18^, many large embayments (e.g., Green Bay, Grand Traverse Bay, Saginaw Bay, the North Channel, Georgian Bay, and the Bay of Quinte), and shoreline types ranging from exposed bedrock to marshy flats to sand dunes^19^. This is further complicated by dynamic wind patterns that vary on decadal, annual, seasonal, and individual storm event temporal scales^20^. Wind patterns even change over the lake during major storm events and can be easily seen in remotely sensed imagery during the winter months (MLIVE; http://www.mlive.com/weather/index.ssf/2017/01/lake_effect_snow_clouds_michig.html). These variable geomorphic and physical factors make the task of accurately representing fetch in the Great Lakes difficult and computationally intensive.

There have been several methods for calculating fetch across lakes. The simplest approaches calculate direct fetch from lake dimensions by using maximum length, a combination of length and width, or lake surface area^21^ commonly resulting in a measurement of the distance across water in the dominant wind direction. This simple measure of fetch can be improved by systematically incorporating the variability of wind patterns. Håkanson^22^ accounted for the variation in dominant wind patterns by averaging the fetch across several wind directions to calculate effective fetch (EF), which is the recommended method by the U.S. Army Corps of Engineers^23^. Keddy^24^ expanded the idea of EF and developed the relative exposure index (REI) that incorporated wind direction and speed. He found that multiplying EF by wind speed (mean or exceedance of 12 mph) yielded better results for predicting shoreline exposure than direct or EF measures alone. We developed two spatially discrete indicators of exposure to provide a more accurate indicator of influence of wind exposure in the nearshore of the Laurentian Great Lakes. We summarized wind data from buoys and used existing tools to calculate EF and REI at a 30-m grid cell resolution. These exposure maps are available for public download for the years 2004–2014.

## Methods

We calculated the EF and REI for the Great Lakes by first summarizing wind data for existing offshore buoys. We then calculated the EF distance for 36 wind directions across 21 lake regions and scaled all the EF distances by the mean wind speed to produce REI. Finally, we developed GIS layers for both wind exposure metrics. Below we describe these steps in more detail.

The first step was to summarize wind data from 21 offshore, moored meteorological buoys operated by the National Oceanic and Atmospheric Administration (NOAA) National Data Buoy Center (NDBC; (http://www.ndbc.noaa.gov/data/historical/stdmet/)) and Fisheries and Oceans Canada (http://www.meds-sdmm.dfo-mpo.gc.ca/isdm-gdsi/waves-vagues/index-eng.htm). These buoys collect real-time wind, wave, and atmospheric weather data during the ice-free season, which is typically from the beginning of April until mid-November. We summarized wind direction frequency and mean wind speed in 36 directions, starting at 0 degrees (north) and proceeding clockwise in 10-degree increments for the years 2004 through 2014. Buoy data were not consistently available, so we set a 50% threshold of ice-free season data availability to include the data in our calculations. Table 1 highlights which buoys and years reported data above this threshold.

**Figure 1:**
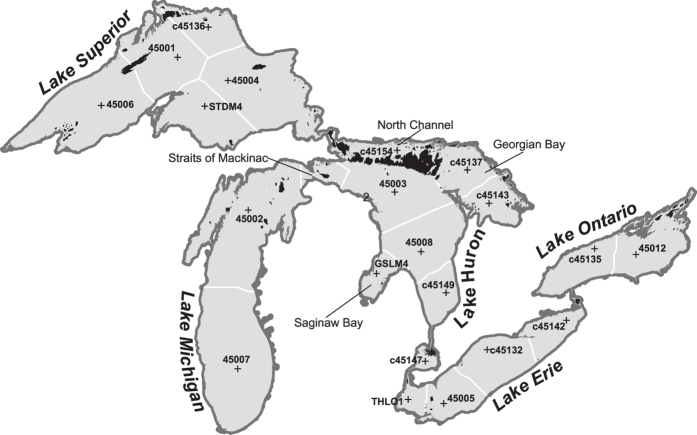
The Great Lakes basin land-water mask showing land in dark gray, islands in black, and water surface in light gray. The offshore buoys used to calculate wind data summaries are shown as “+” marks. White lines designate the exposure/fetch regions used to create the composite effective fetch and relative exposure maps.

To process wind data in GIS, we created a land-water mask separating the five Great Lakes and defining each raster grid cell as land or water based on the location of the center point of the grid cell. We used the Great Lakes Aquatic Habitat Framework^18^ (GLAHF; https://www.glahf.org/) spatial framework of 30-m grid cells covering the entire aquatic areas of the Great Lakes including defined boundaries for shoreline (approximately the ordinary high water mark), basins and sub-basins, bays, and islands^25^. We divided the water surface into regions based on the number and locations of offshore buoys. We mapped offshore buoy locations from the geographic coordinates available from NOAA NDBC and then built Thiessen polygons around the buoys to guide the delineation of fetch regions (Fig. 1). We modified the initial Thiessen polygons to incorporate the following areas of unique wind patterns. For example, the Straits of Mackinac (Fig. 1) connecting Lakes Michigan and Huron are a very energetically dynamic area in the Great Lakes, experiencing strong winds and oscillating circulation patterns^26^ that results in the two lakes hydrologically acting as one. The Straits also have long, straight-line open water paths extending into the northern reaches of both lakes, which influences east-west fetch distances in northern Lakes Michigan and Huron. To incorporate these unique factors into EF and REI calculations, we extended the land-water masks for both lakes to capture the entire Straits of Mackinac into the fetch calculation. We also slightly adjusted the boundaries for three major sheltered bays in the Great Lakes based on wind data: North Channel, Georgian Bay, and Saginaw Bay of Lake Huron. Buoy data exists for these three bays, which are not available for other bays in the Great Lakes.

The second step was to calculate two indices, EF and REI. We calculated the EF distance for each of the 21 lake regions (Fig. 1) and 36 wind directions using methods from the Shore Protection Manual^23^ (SPM). The SPM method estimates EF by calculating the arithmetic mean of nine radial measurements around the desired wind direction at 3-degree increments. Tools designed by Finlayson^27^ implemented the SPM method utilizing ArcGIS for Desktop Toolbox and Python. These tools were last updated by the Upper Midwest Environmental Sciences Center of the U.S. Geological Survey^28^ to operate with the most current version of ArcGIS 10.x. This step resulted in a series of raster files for each wind direction bin and lake region. Each of these directional rasters was then weighted by the frequency that the wind blew in that direction. We calculated the REI by scaling the EF for each lake region (Fig. 1) by mean wind speed. We summarized the mean wind speeds for each of the respective bins and lake regions and multiplied by the direction-weighted EF.

**Figure 2:**
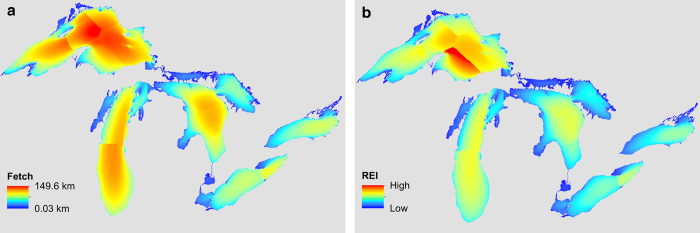
Example maps of effective fetch and relative exposure index. Shown here, for the year 2010, are modeled effective fetch (**a**) and relative exposure index (**b**).

The third and last step was to create the wind composite direction-weighted EF and REI maps for each lake and year by merging them together to create continuous EF and REI raster layers. The EF method produced a continuous layer where each grid cell’s value was the sum of the distance in meters to shore along each input direction weighted by the percent frequency that the wind blew from that direction. The REI method produced a layer where each grid cell’s value was the sum of EF scaled by the average wind velocity for each of the 36 directions. This results in a continuous layer with the units of m^2^ s^-1^; however, the values are treated as dimensionless as the REI method was intended to produce a measure related to the size of the waves arriving on shore integrated over a particular time period. A consequence of defining buoy regions and compositing the EF and REI results were the formation of break lines where regions meet (see Fig. 2). Break lines can be smoothed by using a common image processing technique referred to as a “low pass filter”. A low pass filter averages the values of nearby pixels reducing local variation. This tool is available in ArcGIS, QGIS, R, and many other GIS and image processing software packages.

The completed EF and REI maps are available by lake and year for years 2004–2014 (Table 1), when sufficient data were available. Illustrated in Fig. 2 are example EF and REI maps for the year 2010.

### Code availability

We developed Python scripts to calculate the metrics and develop composite maps. Effective fetch and REI raster layers were developed for each lake region (Fig. 1) using the Python script “wind_weighted_EF_sum_rasters.py”. We created composite maps for each lake and year using the Python script “cost_distance_composite.py”. Both scripts utilize Python version 2.7, ArcGIS for Desktop 10.3, ArcPy module, and the Spatial Analyst Extension. The Python scripts are available as part of the data download package (Data Citation 1) along with an example input wind direction frequency and mean wind speed file.

## Data Records

The newly developed EF and REI maps are available by lake and year as raster layers in GeoTIFF format (Data Citation 1). The files are available for download in zip-archive format. The zip file size is 14.0 GB for the GeoTIFF format, and contains the two Python processing scripts and an example wind summary input file. The file naming convention follows the pattern “lake_method_year”; for example, the EF map for Lake Erie in 2010 is “Erie_EF_2010”. An offshore raster mask named “offshore_mask” has been included representing the lake areas deeper than 30-m. The example wind summary file is called “LSC_c45147_2014_fetchDir_36bins.csv”, where LSC is the initials for Lake St. Clair, c45147 is the buoy name, 2014 is the year, and a brief file description (e.g., fetchDir_36bins.csv). The wind summary file contains three columns, 1) fetch direction (degrees from north); 2) frequency of the fetch direction (%); and 3) mean wind speed (m s^−1^). The EF model uses fetch direction and frequency data and the REI model uses all three data columns.

## Technical Validation

**Figure 3:**
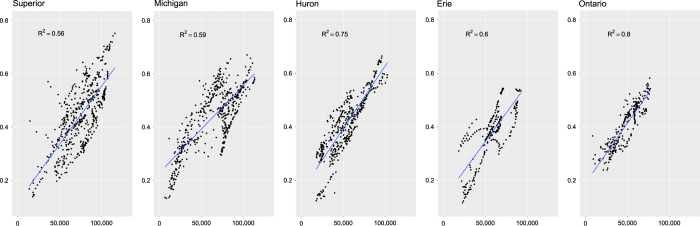
Relationship between mean wave height (m) from the Wave Information Studies and effective fetch distance (m) for each of the five Great Lakes.

To validate the EF model, we compared the output map for each year to the U.S. Army Corps of Engineers Wave Information Studies (WIS; http://wis.usace.army.mil/) wave model mean values by year. The WIS model hindcasts detailed wave information including hourly height, direction, and period for 1,950 locations across the five Great Lakes. The modeled point locations follow the 15 to 20 m depth contours along the entire coastline of the Great Lakes. Since the wave model outputs at such a high temporal resolution (hourly) the data were summarized to capture an annual mean wave height over the same ice-free time period as the EF model, April 1 through November 15 for each of the years 2004–2012. At each of the 1,950 locations, we compared the extracted values from the EF maps to the mean annual wave height by lake and year using linear regression. We found there to be consistent agreement between the output of the two models with a mean R^2^ value of 0.635 (range from 0.329 to 0.813). Figure 3 shows the linear regressions for all lakes for the year 2010.

In a small number of locations (n = 98), we found differences over multiple years between the wave model and EF model results. We identified these locations by applying the linear regression model for each lake and year and tallying the number of times a location was in the upper or lower 5^th^ percentile of the data distribution. The differences between the two models fell into two categories: 1) the wave models underestimated or the EF model overestimated, or 2) the wave models overestimated or the EF model underestimated.

Locations where the WIS model under- or EF model over-estimated were along the western Lake Superior shoreline and three shallow water areas (Green Bay, Saginaw Bay, and southern Lake Michigan). Along the western Lake Superior shoreline, the wave model underestimated due to the underestimation of winds in the Climate Forecast System Reanalysis (CFSR3) product (Robert Jensen, personal communication, 3 November 2017), which is a driving variable for the WIS model.

Consistently, the locations where WIS model over- or the EF model under-estimated occurred around islands and peninsulas, such as northern Lake Michigan, Georgian Bay (Lake Huron), the Keweenaw Peninsula (Lake Superior), Long Point (Lake Erie), and Prince Edward Point (Lake Ontario). The model results for either or both of the models in these locations were most likely affected by the resolution of the land-water masks. The WIS models use a 0.04-degree resolution land mask for Lakes Superior, Michigan, and Huron and a 0.02-degree resolution mask for Lakes Erie and Ontario. The coarse resolution of the WIS land masks misses smaller islands and does not provide a realistic outline for peninsulas. By contrast, the 30-m resolution land mask used for the EF models incorporates the influence of small islands and peninsulas but could exaggerate the wind pattern and underestimate EF. These areas can be smoothed by using the low pass filter method described in the methods section to correct errors due to differences in data resolution.

In summary, the wave model under- or EF over-estimates (n = 62; 3.2%) are capturing known error in the wave models and the wave model over- or EF under-estimates (n = 36; 1.8%) are most likely due to land-water mask resolution. The relationship between mean wave height and REI were similar to those with EF, but the R^2^ values were slightly lower (mean of 0.603; range of 0.250–0.758). This is likely due to the location of buoys in offshore waters (Fig. 1). Offshore wind speeds are likely higher due to lake-atmosphere destabilization, and this could result in an overestimation of the REI in nearshore areas where the wind speeds are typically lower^29^.

## Usage Notes

These new EF and REI layers (Data Citation 1) allow for a consistent, basin-wide representation of wind patterns across the Great Lakes basin. The layers presented cover the entire lake surface, but are most relevant to nearshore and coastal areas. It should be noted, that the wind patterns used for this study were extrapolated from offshore buoy data, which may not replicate the more complex wind patterns along the coastal edge and therefore should be used with caution. Users should also use caution in offshore areas, due to complex open water circulation patterns in the Great Lakes. EF and REI should not be used to estimate thermocline, waves, or currents in the offshore areas. While fetch is a determinant of the size of waves and the depth of the thermocline, Straskraba^30^ found that in large lakes where the fetch is >100 km, the relationship between fetch and thermocline depth is overestimated.

These calculations of EF and REI will be valuable for developing biological models that incorporate coastal and nearshore exposure, identifying areas for restoration or mitigation and estimating relative effects from cumulative wave exposure and varying water levels that contribute to the dynamic coastal habitats of the Great Lakes.

## Additional information

**How to cite this article**: Mason, L. A. *et al*. Effective fetch and relative exposure index maps for the Laurentian Great Lakes. *Sci. Data*. 5:180295 doi: 10.1038/sdata.2018.295 (2018).

**Publisher’s note**: Springer Nature remains neutral with regard to jurisdictional claims in published maps and institutional affiliations.

## Supplementary Material



## Figures and Tables

**Table 1:** Offshore buoy data availability by lake and year, an “X” designates at least 50% of the ice-free season (April–November 15) had collected and available data.

Lake	2004	2005	2006	2007	2008	2009	2010	2011	2012	2013	2014
Erie		X	X			X	X				X
Huron			X	X			X		X		
Michigan	X	X	X	X	X	X	X	X	X	X	X
Ontario					X		X	X	X	X	X
St. Clair			X	X	X	X	X	X	X	X	X
Superior		X	X		X		X				
